# Salient beliefs towards vaginal delivery in pregnant women: A qualitative study from Iran

**DOI:** 10.1186/s12978-016-0120-5

**Published:** 2016-01-23

**Authors:** Parvin Rahnama, Khadigheh Mohammadi, Ali Montazeri

**Affiliations:** 1 Department of Midwifery, Faculty of Nursing and Midwifery, Shahed University, Tehran, Iran; 2 Mental Health Research Group, Health Metrics Research Center, Iranian Institute for Health Sciences Research, ACECR, Tehran, Iran; 3 Faculty of Humanity Sciences, University of Science & Culture, ACECR, Tehran, Iran

**Keywords:** Cesarean section, Theory of Planned Behavior, Normal vaginal delivery

## Abstract

**Background:**

Childbirth by cesarean section has increased at an alarming rate over the past few years in Iran. The present study was designed to explore pregnant women’s beliefs about the mode of delivery in order to provide some suggestions for future interventions to increase vaginal delivery.

**Methods:**

This was a qualitative study framed by the Theory of Planned Behavior conducted in Tehran, Iran in 2013. Pregnant women attending public hospitals were recruited. The data were collected via in-depth interviews and focus group discussions. Interviews were conducted in a semi-structured manner. All interviews were tape recorded and transcribed verbatim. A content analysis approach was used to explore the data.

**Results:**

In all 36 pregnant women participated in the study. The mean age of women was 27.8 (SD = 4.5) years. In general, women preferred vaginal delivery. During interviews and focus group discussions several themes emerged related to the pain associated with vaginal delivery, fears of childbirth, related health concerns, and the role of decision makers. The findings were grouped into three main themes namely: behavioral beliefs (negative and positive beliefs towards outcomes of vaginal delivery), normative beliefs (injunctive norms and descriptive norms), and control beliefs (internal and external barriers).

**Conclusion:**

Despite the fact that there were positive beliefs regarding vaginal delivery, participants indicated concerns related to loss of control and fear. It is essential that health care providers realize the psychological needs of women during pregnancy and the need for continuous support during childbirth. This type of support may improve their self-control during labor, and decrease fear of childbirth.

## Background

The cesarean section rate with no medical indication continues to rise in Iran dramatically. According to the Iranian Demographic Health Survey (IDHS) carried out in October 2000, the rate of cesarean section was reported as high as 35 % [1]; however a recent meta-analysis of 34 studies estimated a cesarean rate of 48 % in Iran [2]. This is an important public health concern as cesarean section is significantly associated with maternal and neonatal morbidity [3–5].

Apart from the medical or obstetrical indications, the choice of mode of delivery may be influence by other factors. Two dominant explanations for these decisions have been raised in the literature: women’s preference for cesarean delivery and service providers’ behaviors [6, 7]. Although a number of studies have highlighted women’s preference for elective cesarean section [8, 9], there is little information regarding women’s beliefs and attitudes towards vaginal delivery [10]. Most existing is quantitative, cross-sectional in nature, and mainly focuses on women’s preference and associated factors derived from statistical analyses [11, 12].

A few qualitative studies looked at women’s beliefs regarding vaginal delivery. For instance, a qualitative study in Argentina of 29 nulliparous women revealed that most women preferred vaginal delivery. The study used a pre-designed guide based on the health belief model and social cognitive theory and revealed that most women preferred vaginal delivery due to cultural, personal, and social factors and it was viewed as normal, healthy, and a natural rite of passage from womanhood to motherhood. The study found that women viewed pain associated with vaginal delivery positively, while they viewed cesarean section as a medical decision [13]. Similarly an investigation from Northern Iran studying 12 pregnant women, 10 women with previous experience of childbirth, seven midwives, seven obstetricians, and nine non-pregnant women and using a ethnographic approach found that cultural beliefs, values and traditions significantly affected individuals’ attitudes towards the mode of delivery, definitions of different modes, and the decisions they made or would make in the future in this regard [14]. Interestingly a study from Sanandaj interviewed 22 Kurdish pregnant women in their third trimester and found that 18 preferred vaginal delivery while four preferred cesarean section. The authors identified four explanations for these preferences: safety of baby, fear of surgery, previous experience and social support [15]. A mixed method study in Tehran using the extended parallel process model (EPPM) reported that there was insufficiently knowledge and wide misconceptions regarding modes of delivery, which influenced the choice of method for delivery. Results of this study also showed that self-confident women were more likely to plan to deliver vaginally [16].

Pregnant women’s beliefs in relation to vaginal delivery are important factor when developing interventions to reduce unnecessary cesarean sections, especially if a theory-driven approach could be applied. Thus, the present study aimed to explore pregnant women’s beliefs regarding vaginal delivery using the Theory of Planned Behavior (TPB) as a framework.

The Theory of Planned Behavior is a social cognitive model of decision-making that provides a useful framework for explaining health behaviors [17, 18]. The TPB suggests the central determinant of behavior is the *intention* to perform a given behavior. The three core components to predict intention according to TPB are attitude, subjective norms and perceived behavioral control (PBC).

The attitude refers to behavioral beliefs about a particular outcome. The subjective norms refers to whether it is believed that others will approve or disapprove of the behavior Perceived behavioral control refers to beliefs surrounding factors likely to facilitate or inhibit the behavior [17, 18].

## Methods

### Design and data collection

This was a qualitative study framed by the Theory of Planned Behavior in Tehran, Iran in 2013. The data were collected using in-depth interviews and focus group discussions with pregnant women. Participants were recruited from pregnant women attending public hospitals willing to participate. Recruitment of participants for the in-depth interviews (*n* = 16) and the focus group discussions (*n* = 20) were based on purposeful sampling. Pregnant women were included if they were: 18–35 years old; over 32 weeks of gestational age; without complication during pregnancy; and without preexisting medical illness. In-depth interviews were conducted in a semi-structured manner. Interviews focused on the perceived outcomes of vaginal delivery. The outcomes included the woman’s positive or negative beliefs of having a vaginal delivery. Also, we asked women to indicate how other people, who may be in some ways important to them, would approve or disapprove of vaginal delivery. Finally women were asked about factors that might inhibit or facilitate vaginal delivery. The duration of interviews ranged from 35 to 45 min. The data collection was continued until data were considered saturated and no new data were emerging. Also two focus group discussions consisting of 10 pregnant women each (*n* = 20) were held. The focus group discussions had an open and semi-structured contradict each other; either it was unstructured or semi-structured, each lasting approximately 60 to 70 min, and began with a few general questions to initiate conversation. Focus group facilitators used probes as necessary to provoke discussions. Participants were encouraged to talk freely and not to hesitate in sharing their experience and feelings.

All in-depth interviews and focus group discussions were tape-recorded, transcribed verbatim, and analyzed subsequently. PR (a female midwife and doctorate in health education) and KM (a female midwife and reproductive health expert) both with experience in conducting qualitative studies carried out interviews and focus group discussions. There was no relationship between interviewers and participants. All interviews and focus group discussions were carried out in a private setting in the hospitals.

### Data analysis

**Fig. 1:**
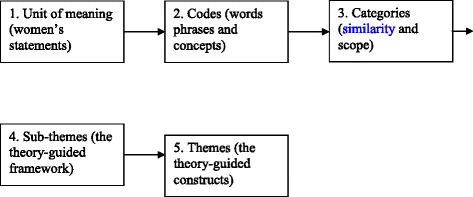
A schematic relationship between the codes, categories, sub-themes, and themes

The transcripts were read, re-read and analyzed separately by two researchers. A content analysis approach was used to derive codes, categories, sub-themes, and themes from the data. Transcripts were read several times to gain primary perception and identify the initial codes or meaning units. The combinations of related initial codes were labeled to form categories and sub-themes. Finally, the main themes were extracted. All analyses were performed by hand. A schematic relationship between the codes, categories, sub-themes, and themes is presented in Fig. 1.

### Ethics

The ethics committee of Shahed University approved the study. We obtained written informed consent from participants after comprehensive explanation of procedure involved.

## Results

In all, 36 pregnant women participated in this study. The mean age of pregnant women was 27.8 (SD = 4.5) years. The characteristics of participants are shown in Table 1.

**Table 1:** The characteristics of the study sample

	Number	(%)
Age (years)		
18-30	26	72.2
31-35	10	27.8
Women’s Education		
Primary	3	8.3
Secondary	22	61.1
Higher	11	30.6
Employment status		
House wife	30	83.3
Employed	6	16.7
Husband’s Education		
Primary	5	13.9
Secondary	16	44.4
Higher	15	41.7
Gravidity		
1	15	41.7
2-5	21	58.3
Previous method of delivery		
None	18	50
Vaginal delivery	6	16.7
Cesarean section	12	33.3
Insurance		
Yes	29	80.6
No	7	19.4

Overall three themes and six sub-themes emerged from the analysis. As shown in Table 2 we framed these under three constructs derived from the theory of planned behavior: behavioral beliefs (negative and positive beliefs towards outcomes of vaginal delivery), normative beliefs (injunctive norms and descriptive norms), and control beliefs (internal and external barriers). During interviews and focus group discussions several topics including issues related to pain due to vaginal delivery, fear of childbirth, some health concerns and discussion about people who might influence women’s decision for selecting the mode of delivery received more attention. Thus the findings, as much as possible, were organized under original themes covering the above-mentioned topics.

**Table 2:** Themes and sub-themes

Theme	Sub-theme
Behavioral beliefs	
	Positive behavioral beliefs
	Negative behavioral beliefs
Normative beliefs	
	Injunctive norms
	Descriptive norms
Control beliefs	
	Internal barriers
	External barriers

### Behavioral beliefs

#### Positive behavioral beliefs

Most participants believed that the vaginal delivery was a natural and normal method and considered it as God’s opinion. Such expressions in a society where religion plays an important role were not surprising; however, a number of other benefits of vaginal delivery were described. Participants stated that vaginal delivery is a physiologic, normal and traditional event. They stated that in the past, lay midwives were responsible for delivery and caring pregnant women during and after childbirth, primarily based on experience and knowledge acquired informally. One woman described that cesarean sections were very rare and it is said that the Iranian hero ‘Rostam’ was the first born by cesarean.

*Rostam’s mother was Rudaba. Rudaba’s labor of Rostam was prolonged due to the extraordinary size of her baby. Zal her lover and husband, was certain that his wife would die in labor. Rudaba was near death when Zal decided to summon the Simurgh (a benevolent, mythical flying creature). The Simurgh appeared and instructed him upon how to perform a ‘Rostamzad’ or ‘Rostamineh’ (Persian equivalent for cesarean section), thus saving Rudaba and the child* [19].

A woman with previous childbirth experience said that:

*Generally I want to say that I think natural delivery is the best way since it is the traditional way. I think we should not be frightening pregnant women saying that normal delivery has pain. Most young women say they are afraid of natural delivery because they feel they might suffer from a lot of pain. (32-old-year, secondary education, housewife, gravidity 2)*

Most interviewees knew that there would be pain relief and rapid recovery immediately after giving birth vaginally. A woman referred to a friend and described:

*A friend said that she would never regret for vaginal delivery. She was telling me that she never suffered from wound and scar. She said that you only experience pain for some hours and then a weak after you are yourself and could do your chores (25-old-year, secondary education, housewife, gravidity 1)*

Another participant said that:

*In natural delivery you experience pain only for hours, just for an hour or less; then you will forget everything. (26-old-year, higher education, employed, gravidity 2)*

Health benefits were another reason some women preferred vaginal delivery. A woman said that:

*This is the pain of natural delivery that brings health. Such pain does not matter since cesarean deliveries bring so many problems to women. (29-old-year, higher education, employed, gravidity 2)*

Also they believed that the normal vaginal delivery is part of being a mother. Some women indicated that motherhood only could be achieved if you experience natural delivery.

*I was ready to tolerate pain but instead do natural delivery because I knew that after one week the pain would gone and I would enjoy my delivery. I believe in ‘no pain, no gain’ idiom. Suffering is needed to become a mother right from a beginning (26-old-year, secondary education, housewife, gravidity 2)*

Although all participants mentioned that vaginal delivery was associated with severe pain, they indicated advantages of tolerating pain during childbirth. Most importantly we found a reflection of religious beliefs for this as a women said that:

*My preference for normal delivery is that I believe God had some good reasons for vaginal delivery. It seems that there should be some positive hidden reasons for mother and baby in natural delivery. When a woman tolerates pain in natural delivery, her sin will be forgiven. This is why it is said that the paradise is under mothers’ feet. (35-old-year, secondary education, housewife, gravidity 1)*

Women attributed health effects to vaginal delivery including exclusion of dirty blood and infectious content of uterus immediately after childbirth.

*Relatives say that in natural delivery everything including blood and infectious materials in the womb will come out, but in cesarean the infectious materials will lessen slowly through afterward bleeding. Generally, it is believed that natural delivery is a better fit to our body. (27-old-year, secondary education, housewife, gravidity 2)*

Furthermore, they believed that during the process of vaginal delivery the hormones secreted are necessary for their health.

*The body of a woman who receives cesarean delivery does not secrete certain hormones and consequently she gets several problems but in natural delivery the body would secrete the hormones that are necessary for women’s health. (28-old-year, secondary education, housewife, gravidity 3)*

#### Negative behavioral beliefs

Women identified several disadvantages of vaginal delivery. Fear was mentioned as an important psychological influencing preferences fear of pain, fear of fetal birth injuries, and fear of the unexpected delivery date.

*I was always worried that during vaginal delivery my baby to be harmed. So I have decided to have cesarean section. (35-old-year, higher education, employed, gravidity 1)*

Health concerns that women expressed were diverse and included incontinence, pelvic infection, and pelvic organ prolapse, and sexual dysfunction. For instance a participant pointed out that:

*I think the womb will lose its original form. Thus I do not like to have normal delivery. Yes, it is good to have a normal delivery but I do like to keep my shape. (29-old-year, secondary education, employed, gravidity 1)*

Other perceived disadvantages of vaginal delivery related to the labor and delivery process: embarrassment, discomfort during the vaginal exam, and the body position required. In addition, participants reported some problems that might occur during labor that would make cesarean section necessary.

### Normative beliefs

#### Injunctive Norms

Injunctive norms refer to behaviors that are perceived as being approved or disapproved of by other people. Such norms typically assist an individual in determining what is acceptable and what is unacceptable social behavior.

Almost all participants considered physicians and health care providers to be the most influential people in their decision about the mode of delivery.

*I only listen to my doctor’s advice. I don’t care about other people’s sayings. If he tells me that you should have cesarean delivery I will accept. (23-old-year, higher education, employed, gravidity 1)*

Another woman said that:

*Natural delivery has less bleeding. I have investigated about it beforehand, but unfortunately my doctor doesn’t let me to do it. (34-old-year, higher education, employed, gravidity 2)*

Similarly a woman said that:

*Most doctors say we do not do natural delivery. They say we don’t have enough time. These doctors try to convince women to do cesarean because they want to get more money. I believe first we should focus on doctors rather than women if we wish to increase natural delivery. (24-old-year, primary education, housewife, gravidity 1)*

Another woman told that:

*If I could decide about it I certainly would choose natural delivery, as I will be fine very soon. My doctor rejects natural delivery. He says it is better not to do natural delivery. Most doctors are in favor of cesarean section. (26-old-year, primary education, housewife, gravidity 2)*

Additionally, friends and family members were frequently identified as most important people that influence women’s decision:

*My sisters say that the natural delivery is better since you can keep your baby better. You can leave the bed very soon but in cesarean section you should lay in bed for sometime as you have pain. (24-old-year, secondary education, housewife, gravidity 1)*

Likewise, one participant said that:

*Most of the time my mother tells me that do the natural delivery. She gave birth to four children. I saw my sister in law and also my neighbor that had cesarean section. Both of them had post-delivery problems. (22-old-year, primary education, housewife, gravidity 2)*

The majority of participants believed that their husbands prefer vaginal delivery. When we asked why their husbands prefer vaginal delivery, women indicated that they think vaginal delivery is a safer method for mother and her baby:

*My husband heard that natural delivery is better. He believes it is healthier and has fewer problems. He says that do the natural delivery. (24-old-year, secondary education, housewife, gravidity 1)*

#### Descriptive Norms

Descriptive norms involve perceptions of behaviors that are typically performed in a community. They normally refer to the perception of others’ behavior.

*My sister had vaginal delivery and I am going to have vaginal delivery too. I think there would be fewer complications after vaginal delivery as many women around me say so. (28-old-year, primary education, housewife, gravidity 1)*

Similarly a participant stated that:

*My sister says that natural delivery is better as you can take care of your baby better. You can leave the bed very soon but in cesarean delivery you should be on bed rest for a long time as you will have pain. (26-old-year, higher education, housewife, gravidity 1)*

### Control beliefs

Control beliefs refer to an individual’s perception of their ability to perform a behavior and can be influenced by internal and external factors. The study participants stated several internal and external barriers for vaginal delivery. Pregnant women considered inadequate mental and physical strength as internal barriers for vaginal delivery. Another internal barrier associated with vaginal delivery was lack of self-confidence.

*I believe that for vaginal delivery one should be strong enough. I am incapable to do so since I am so fragile. (26-old-year, secondary education, housewife, gravidity 2)*

Poor quality care for women and their children during labor was the most commonly cited external barrier for vaginal delivery.

*If those who are present in labor room help pregnant women and give some clue then one would feel much better for normal delivery (30-old-year, secondary education, housewife, gravidity 5)*

Some participants indicated that when health care providers informed them about vaginal delivery and its health consequences, they were more likely to choose vaginal delivery.

*I came here for my first delivery. They provided us a short lecture on normal delivery and its advantages including the fact that normal delivery does need anesthesia and the mother and child will not experience any harm. You should just bear a little pain.’ (22-old-year, secondary education, housewife, gravidity 1)*

### Perceptions of women with and without previous childbirth experience

We carried out a re-analysis of the data in order to see if there were differences in perceptions between women with and without childbirth experiences. It appeared that women with previous childbirth were more likely to be in favor of vaginal delivery while those without previous childbirth experience were more likely to be in favor of cesarean section. A summary of findings is presented in Table 3.

**Table 3:** A summary of expressed beliefs by women with and without previous childbirth experience

	With previous childbirth experience	Without previous childbirth experience
Pain	Expressed positive attitudes towards pain tolerance during vaginal delivery	Expressed worries about pain for vaginal delivery
Fear	No fear expressed	Expressed fear of fetal injury, and unexpected delivery date
Main desire	Experience of a real motherhood	Like to keep body shape
Embarrassment and discomfort	Not mentioned	Mentioned embarrassment and discomfort with vaginal delivery
Views on mode of delivery	Viewed vaginal delivery as natural and normal	Viewed vaginal delivery as suffering
Overall expressed preference for mode of delivery	Vaginal delivery	Cesarean section

## Discussion

This study explored pregnant women’s beliefs related to vaginal delivery and found a tendency to prefer vaginal delivery. We also found that a range of behavioral, normative and control beliefs were involved in shaping women’s decision on selecting the mode of delivery. Exploration of salient beliefs towards revealed a number of underlying factors that might influence women’s decisions and should be taken into account when designing interventions for promoting vaginal delivery. Recent evidence examined interventions designed to increase vaginal delivery, although inconsistent, suggest that educational strategies delivered by opinion leaders would significantly increase vaginal delivery [20, 21]. In Iran where the majority of women are Muslim (98 %), religious leaders could play an important role. In addition as suggested Iranian midwives could help increase vaginal delivery if they learned more about normal birth and evidence-based care, and to gain confidence in their ability to facilitate birth [22].

Fear of childbirth was one of the psychological factors that contributed to women’s decision in preferring cesarean section. Such observation implies that prenatal care educational programs should be implemented, and should include discussion regarding the fears of childbirth. Studies have shown that childbirth related fear was among the most common reasons for preference for cesarean section [23, 24].

Results showed that participants were concerned about the pain of vaginal delivery; some were positive and some were negative. However, we noted that the positive aspects of pain were greater than those that were negative. Women with positive attitudes believed that pain should be tolerated to gain rewards including moving from womanhood to motherhood. Studies reported that the pain during childbirth was often seen as a confirmation of passage into motherhood, natural and worth suffering [13, 25].

Women reported that health care providers encouraged them to have cesarean section. Similarly a qualitative study of 14 women in Australia indicated that women who had caesarean section in their first pregnancy in the absence of a known medical indication perceived that their preference for cesarean was safe and responsible [26]. A systematic review of quantitative studies that evaluated non-clinical interventions for increasing the uptake of vaginal birth indicated that national guidelines influenced vaginal birth rates, but a greater effect was seen when institutions developed local guidelines, adopted a conservative approach to caesarean section, used opinion leaders, gave individualized information to women, and gave feedback to obstetricians about mode of birth rates [27]. However, as a quasi-experimental study from Iran reported, even applying clinical practice guidelines alone could not guarantee that the cesarean section rate would decrease [28], perhaps a more effective way is to engage women in the process of decision-making. A qualitative study from UK found that many women did not participate actively in the decision-making since they felt uncomfortable with the responsibility. This study also found that feelings about the amount and quality of the information received regarding delivery options from health care providers varied greatly, with many women wishing for information to be tailored to their individual clinical circumstances and needs [29].

The present study clearly demonstrates that pregnant women mentioned a number of barriers regarding vaginal delivery including internal and external barriers. Studies have shown that pregnant women feared their own abilities related to vaginal delivery or expressed fear of losing control during delivery [30, 31]. It seems that the increase of personal confidence for vaginal delivery would decrease fears and thus vaginal delivery would decrease fear. A recent publication highlighted association between *strength of preference* for vaginal delivery and likelihood of vaginal birth [32]. The findings of this study also indicated that there were a number of external barriers that influenced women’s preferences. These factors included issues related to insufficient or inappropriate care during labor and inadequate relationship with their health providers to gain accurate information in prenatal care. Studies have shown that women during childbirth need continuous support for a good birth experience [33, 34]. It was suggested that women who receive continuous support during childbirth by a midwife is likely to feel less alone and restoring the woman’s trust in herself [35].

Finally as it relates to the theoretical framework of the study, one might argue that if we hope to reduce unnecessary cesarean sections and positively impact women’s choice for vaginal delivery, there is need to reinforce positive behavioral beliefs among women when attending antenatal care units. We should encourage women and indicate that they have enough strength for vaginal delivery. At the same time we need to tackle internal and external barriers both within and outside families. As indicated in this study physicians and health care providers have important role in shaping women’s decision. Thus, they should also be trained through regulations and standard audits.

### Strengths and limitations

A major strength of our study was the fact that we used a qualitative method to investigate salient beliefs towards vaginal delivery in pregnant women. However, our results were derived from a sample of women living in Tehran and thus, it may not be generalized to all women living in Iran. Finally it is worth noting that since this study focused on vaginal delivery, it would not add to the literature on women’s choice for elective cesarean section.

## Conclusion

Although findings revealed that most pregnant women had positive beliefs towards vaginal delivery, it was found that pregnant women need more guidance by midwives and obstetricians. Irrespective of the positive beliefs, health care providers should address women’s psychological needs during pregnancy and give continuous support during childbirth. These strategies could improve their self-control during labor, decrease fears of childbirth and increase satisfaction with their birth.
